# GINIplus and LISAplus – Design and selected results of two German birth cohorts about natural course of atopic diseases and their determinants

**DOI:** 10.5414/ALX01455E

**Published:** 2017-08-04

**Authors:** J. Heinrich, I. Brüske, C. Cramer, U. Hoffmann, M. Schnappinger, B. Schaaf, A. von Berg, D. Berdel, U. Krämer, I. Lehmann, O. Herbarth, M. Borte, A. Grübl, C.P. Bauer, C. Beckmann, H. Behrendt, J. Ring, S. Koletzko

**Affiliations:** 1 Institut für Epidemiologie I, Helmholtz Zentrum München, Deutsches Forschungszentrum für Umwelt und Gesundheit, Neuherberg; 2 Leibniz-Institut für Umweltmedizinische Forschung (IUF), Düsseldorf; 3 Kinderklinik, Technische Universität München; 4 Praxis für Kinder- und Jugendmedizin, Bad Honnef; 5 Forschungsinstitut, Klinik für Kinder- und Jugendmedizin, Marien-Hospital, Wesel; 6 Department Expositionsforschung und Epidemiologie, UFZ – Helmholtz Zentrum, Leipzig; 7 Institut für Umweltmedizin und Hygiene, Medizinische Fakultät, Universität Leipzig; 8 Kinderklinik, Städtisches Klinikum St. Georg, Leipzig; 9 Zentrum Allergie und Umwelt (ZAUM), Technische Universität und Helmholtz Zentrum München; 10 Christine Kühne Center of Allergy Research and Education (CK-CARE), Klinik und Poliklinik für Dermatologie und Allergologie, Technische Universität München; 11 Dr. von Hauner‘sches Kinderspital, Ludwig-Maximilian-Universität München, Germany

**Keywords:** allergy, East-West-Germany, lifestyle, environmental factors, genes, epidemiology, children

## Abstract

The increasing prevalence of asthma, hay fever, and allergic sensitization in Western Germany after east-west division in 1949 and their rapid increase in East German children after re-unification in 1990 are strong indications for the role of life-style and/or environmental factors in the development of atopic diseases. Obviously, the perinatal period is crucial for priming the immune system. Therefore, explorations of determinants of atopic diseases need pregnancy or birth cohorts as the most appropriate epidemiological study designs. This review presents the design and selected results of the two German birth cohorts GINIplus and LISAplus. GINIplus and LISAplus recruited 5.991 and 3.097 healthy, term newborns, respectively, from Munich, Wesel, Leipzig, and Bad Honnef. Approximately 55% could be followed for the first 10 years. We analyzed the natural course of atopic diseases and the role of life-style, environmental, and genetic factors for disease onset, intermediate phenotypes, and genes involved in detoxification and oxidative stress. The results of these two large birth cohorts contributed substantially to the understanding of atopic diseases and their determinants.

German version published in Allergologie, Vol. 35, No. 1/2012, pp. 20-31

## Introduction 

The development of atopic diseases, such as eczema, hay fever, and asthma, has been understood to be the consequence of a complex interaction of environmental factors, familial or genetic predisposition, and – recently also – epigenetic phenomena. While it is certainly justified that the genetic component be thoroughly investigated, it was wrong to neglect investigations on the role of environmental factors, including lifestyle-dependent factors. 

The division and re-unification of Germany provides a unique setting to illustrate the outstanding role of lifestyle-dependent factors for the etiology of allergic diseases. At the beginning of the 1990s, the prevalence of asthma, allergic rhinitis, and allergic sensitization in Western Germany were markedly higher than in Eastern Germany [1, 2]. 

This difference could mainly be seen in people born after 1960, while in people born before 1955 the prevalence was similar in both Eastern and Western Germany [3, 4]. As the populations of Eastern and Western Germany are comparable, the higher prevalence in the West was attributed to the “Western lifestyle” [5, 6]. Indeed, the number of allergic diseases in Eastern Germany increased significantly in the years after re-unification [7, 8]. The difference in the prevalence of allergic sensitization and hay fever, which was significant at the beginning of the 1990s, had diminished by the year 2000 [9]. 15 years after re-unification, the national health survey in children and adolescents, which included almost 18,000 children and adolescents, showed no differences between Eastern and Western Germany with regard to asthma, hay fever, or allergic sensitization [10]. The “Western lifestyle” influences many aspects of daily life, like for example housing (indoor allergens and chemicals, air exchange) and living environment (air pollution due to living near busy streets), nutrition (breast-feeding, introduction of solid food, hypoallergenic food, fatty acids in nutrition, antioxidants), medical care (vaccinations, increasing numbers of Caesarean sections, antibiotics), child care (age of onset of group care), traveling (contact with new allergens), and number of children per family (the more children per family the more infections; infections brought in by older siblings). These environmental and lifestyle factors seem to play a major role in the peri- and post-natal period when the immune system is developing [11]. 

The two large German birth cohorts, GINIplus and LISAplus, recruited 5,991 and 3,097 children, respectively, in Munich (West), Leipzig (East), Wesel (West), and Bad Honnef (West) to investigate various influences on the development of the immune system and the etiology of allergic diseases. The design of these studies and a summary of selected results are presented here. 

## Design of GINIplus and LISAplus 

The study design of both studies is almost identical. Details are presented in the following paragraphs. 

### GINIplus – The German Infant Study on the Influence of Nutrition Intervention plus Air pollution and Genetics on Allergy Development

GINIplus is a prospective population-based birth cohort study. Healthy, mature newborns with German parents and a birth weight of more than 2,500 g were recruited between September 1, 1995 and October 31, 1997 (Munich) or until June 30, 1998 (Wesel) in obstetric hospitals in Munich (10 hospitals) and Wesel (8 hospitals). Newborns whose mothers suffered from an immunologically relevant chronic disease, like diabetes, HIV, or autoimmune disease, or who were drug or alcohol addicted, were excluded. Children whose parents were not sufficiently capable of the German language were also not included in the study. Furthermore, newborns were excluded if their families lived more than 50 km from the city center or planned to move away from the study region. GINIplus consists of two study arms with specific aims: “German Infant Nutritional Intervention Study (GINI study)” and a non-interventional study. Both study arms are complementary and are based on identical recruiting of newborns in the same hospitals at the same time. 

**Figure 1.**
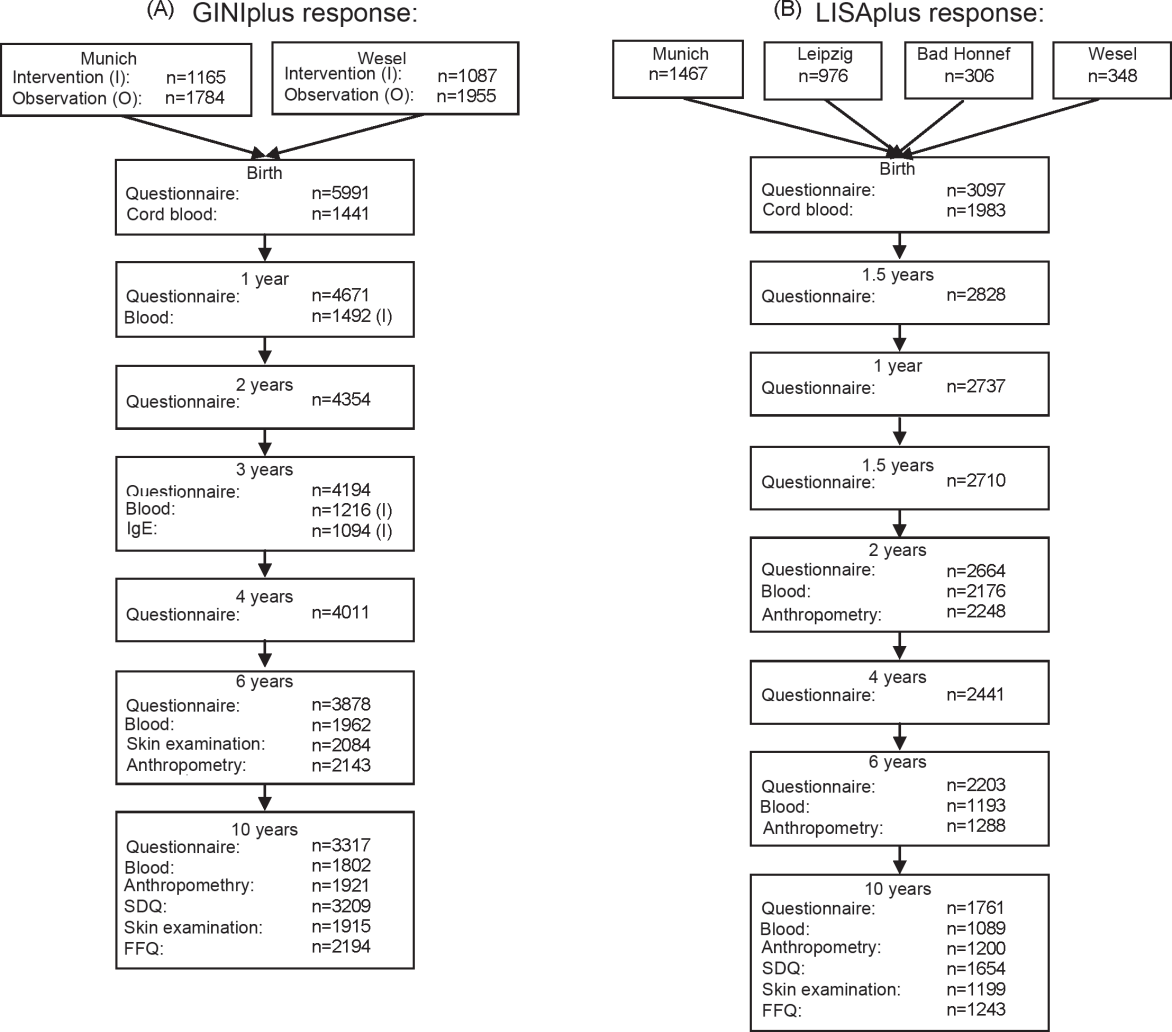
Study structure and response of GINIplus (A) and LISAplus (B).

The interventional arm of the GINIplus study was a double-blind, randomized, placebo-controlled trial (DBRCT) using hydrolyzed formula nutrition and carried out in children with a family history of allergy during the first 4 months of life. The design of this trial and the results are presented in another article in this issue (von Berg et al. p. 32-43). Children without a family history of allergic diseases (no asthma, hay fever, eczema, food allergy or allergic urticaria present in father, mother, or siblings) and children with such a family history whose parents did not want them to participate in the study or lived outside the studied region and therefore could not be recruited for the interventional arm were observed in the non-interventional study arm. The following study intervals were used in the GINIplus study: birth, Year 1, 2, 3, 4, 6, and 10 as well as the ongoing 15-year follow-up (Figure 1). Of the 5,991 newborns recruited, 3,317 (55.4%) could be followed until the age of 10 years. The items investigated in each interval are displayed in Table 1. 

Furthermore, the items investigated in the ongoing 15-year follow-up can be seen in detail on the Internet: www.ginistudie.de 

### LISAplus – Influence of Life-style factors on Development of the Immune System and Allergies in East and West Germany plus Air Pollution and Genetics on Allergy Development

3,097 healthy, mature newborns with German parents and a birth weight > 2,500g who were born between November 11, 1997 (Munich, Leipzig, Bad Honnef) or July 1, 1998 (Wesel) and January 31, 1999 were included in the LISAplus study. Exclusion criteria were the same as in the GINIplus study. The subjects were recruited in 4 obstetric hospitals in Leipzig (976 children), in 6 hospitals in Munich (1,467), in 3 hospitals in Wesel (348), and 1 in Bad Honnef (306) by asking the mothers of the eligible newborns after they had given birth. Approximately 55% of eligible families participated. The following survey intervals were applied: birth, 3 months, 6 months, 1 year, 1.5, 2, 4, 6, and 10 years. At the age of 2, 6, and 10 years, the children were medically examined (Figure 1). 

Of the 3,097 recruited newborns, 1,760 (56.8%) children could be followed until the age of 10 years. 

The items investigated are presented in Table 1. 

At birth, pregnancy history, social factors, and family history of allergic diseases were collected. As markers of immunocompetence, total immunoglobulin E (IgE), and in a subgroup also the cytokine production in the peripheral T-lymphocytes, were determined in the cord blood. In 269 children, the amount of volatile organic compounds (VOC) was measured in the first 4 weeks of life and at the age of 13 months. To determine the endotoxin and allergen exposure, dust was collected from the homes of all Munich and Leipzig participants when the children were 3 months old, and the housing situation was assessed. 

During the first year of life, the parents provided monthly protocols to assess the health-related development and the nutrition of the children; in addition, every 6 months, questionnaires were filled in until the child was 2 years old, followed by further questionnaires at the age of 4, 6, and 10 years. The questionnaires assessed data on various exposure factors, housing situation, nutrition, and contact with pathogens and allergens. Furthermore, data on allergic diseases and infections were collected. At the age of 2 years, blood was drawn from all participants to determine specific IgE, IgG subclasses, vaccination antibodies, and viral IgG antibodies; in a subgroup of participants, cytokine production of the peripheral T cells were measured again. At the ages of 6 and 10 years, IgE measurements were repeated, and blood was drawn for genetic analyses. In addition, the skin was examined at ages 6 and 10, and if clinical signs of atopic eczema were detected, a skin prick test was carried out. At the age of 10, a broad spectrum of inflammation markers were determined. 

## Selected results 

### Natural course of atopic diseases

**Figure 2.**
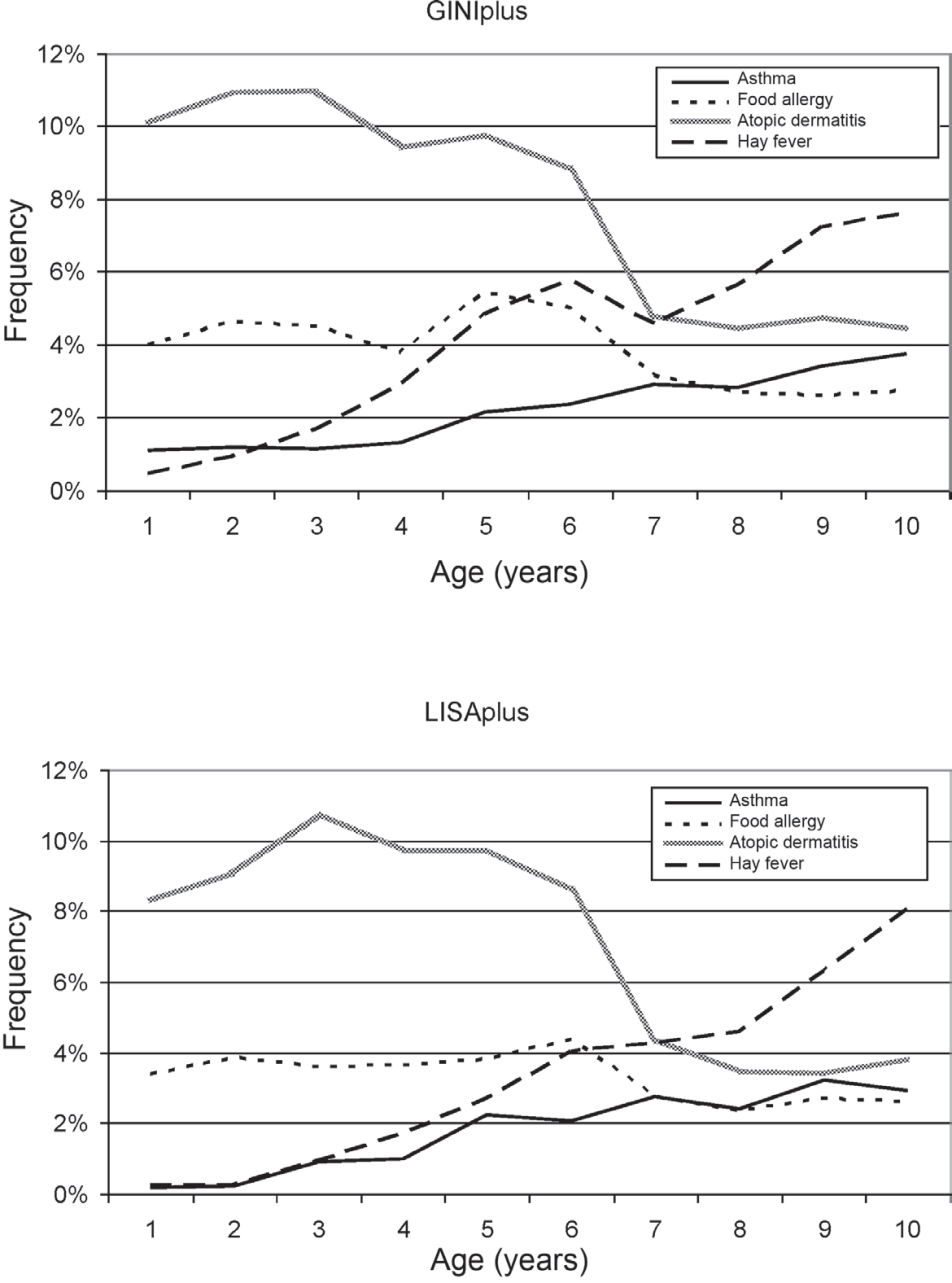
Age-related course of allergic diseases during the first 10 years of life of 5,991 (GINIplus cohort) and 3,097 (LISAplus cohort) children.

Figure 2 shows the courses of asthma, food allergies, eczema, and hay fever according to diagnoses made by physicians and communicated by the children’s parents. Eczema and food allergies become less with increasing age, while asthma and hay fever are more frequent in older children. 

**Figure 3.**
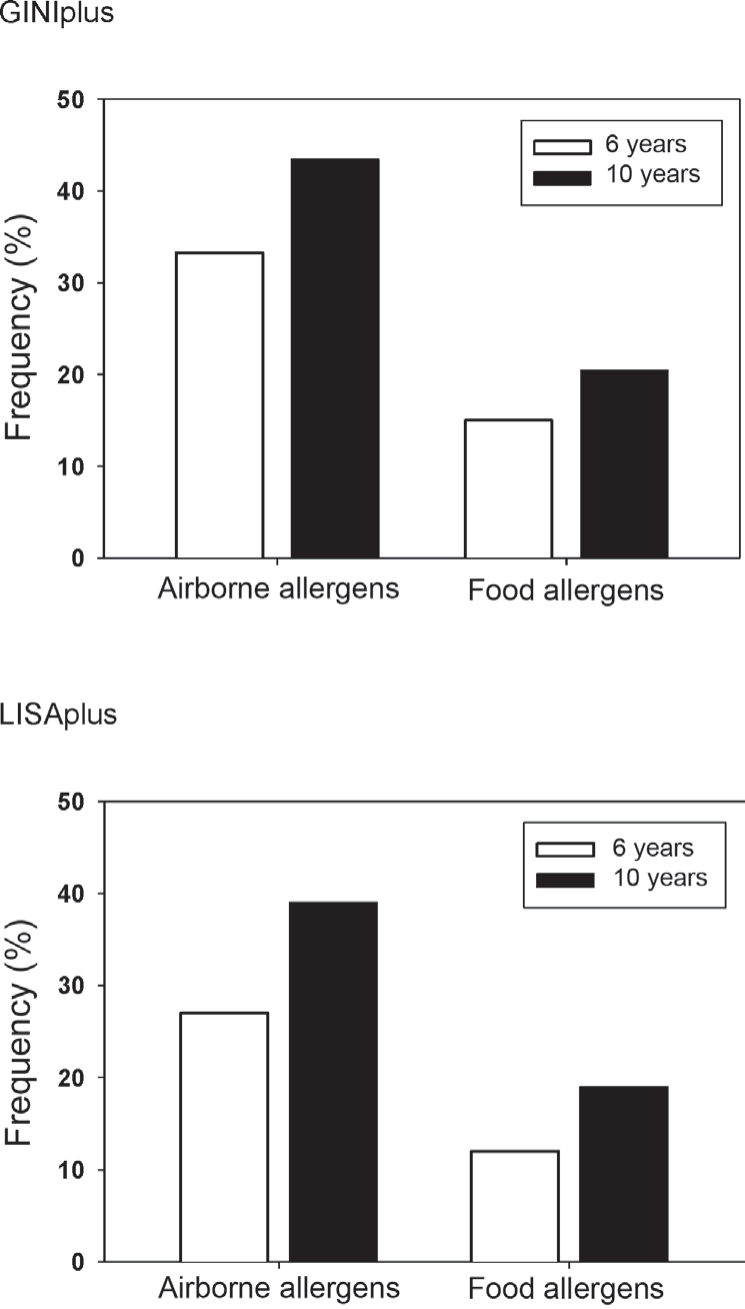
Age-related increase in allergic sensitization from the 6^th^ to the 10^th^ year of life in GINIplus and LISAplus children (RAST). Airborne allergens: grass, birch, mugwort, rye, Cladosporium herbarum, house dust mite, dog, and cat allergens. Food allergens: egg, cow’s milk, fish, wheat, peanut, soy.

Figure 3 shows a marked increase of allergic sensitization against airborne allergens, but also against food allergens from the 6^th^ to the 10^th^ year of life. At the age of 10, almost half of the children react positively to airborne allergens. 

### Regional differences in atopic diseases

**Figure 4.**
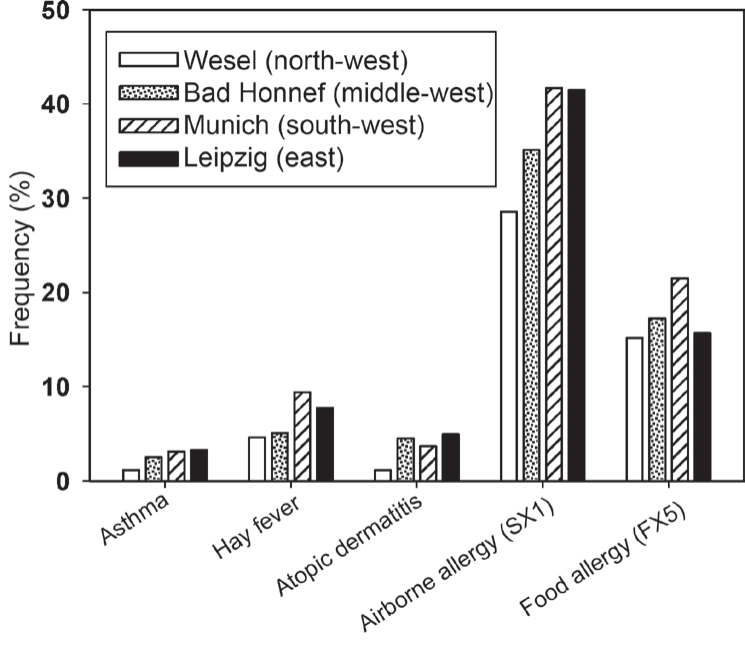
Regional differences in the frequency of atopic diseases and allergic sensitization in 10-year-old children. Results of the GINIplus and LISAplus studies (combined).

Figure 4 shows marked regional differences with regard to the frequency of asthma, hay fever, eczema, and allergic sensitization against airborne allergens (sx1) and food allergens (fx5). All atopic diseases and rates of allergic sensitization are lower in the rather rural area of Wesel as compared to the urban study regions. When the two cities Leipzig (Eastern Germany) and Munich (Western Germany) are compared today, the differences are not as pronounced as they were in the epidemiological studies of the early 1990s [2]. Similar to the sample of the health survey in children and adolescents [10], which is representative for all of Germany, GINIplus and LISAplus do not show differences between Eastern and Western Germany with regard to the prevalence of asthma, hay fever, or allergic sensitization. 

Several studies demonstrated that the prevalence of eczema has slightly different regional patterns when the Eastern and the Western parts of Germany are compared. Eczema was more frequently observed in Eastern Germany [12, 13, 14]. This was also reflected in LISAplus: Until their 6^th^ year of life, children from Leipzig had a higher annual prevalence and cumulative incidences of eczema than their peers in Western Germany [15]. Among numerous risk factors with regionally different frequencies, whether children started nursery school earlier (before their 2^nd^ birthday) could be identified as an explaining factor for the differences between the East and the West. In Leipzig, far more children attended nursery school from that early age, as compared to the Western German study locations, and furthermore, attending nursery school was more frequently associated with eczema in Leipzig. To what extent this association was causal could not be answered. 

### Allergic sensitization in the 1^st^ year of life is a risk factor for the development of allergic diseases later in life

10.9% of GINI children (with family history of allergy) experienced allergic sensitization, i.e., IgE against an airborne or food allergen (mite, cat, timothy grass, birch pollen, cow’s milk, egg, soy), during their 1^st^ year of life. Five years later, these children were more prone to have allergic diseases than the other 6-year-olds who had not yet experienced allergic sensitization. More than twice as many of the children who were sensitized at the age of 1 had an eczema later (20.6% vs. 9.4%); for hay fever it was also more than twice as many (15.4% vs. 7.3%); and asthma was diagnosed almost 4 times as often in children who experienced sensitization during their 1^st^ year of life (10.2% vs. 2.6%). Eczema manifested mainly due to sensitization against cat, milk, or egg. Sensitization against grass pollen was associated with a high probability of developing asthma or hay fever later [16]. The joint analysis of GINIplus and LISAplus data on allergic sensitization against food allergens in early childhood showed a statistically significant increase of the risks of developing asthma and eczema during the first 10 years of life [17]. 

### Genetic analyses

Data derived from GINIplus and LISAplus were included in the population-based evaluation within a large international meta-analysis comprising 5,606 cases of eczema and 20,565 controls [18]. This study identified three new genes (OVOL1, ACTL9, KIF3A) that are associated with eczema. These genes are involved in the regulation of epidermal proliferation and cell differentiation as well as in cytokine secretion and thus suggest possible biological mechanisms that contribute to eczema development. 

A genetic variant of the high-affinity receptor for IgE (FCER1A) is clearly associated with total IgE. In GINIplus and LISAplus, we found that this association with IgE is the same at birth (cord serum) and at the age of 2, 3, and 6 years [19]. Obviously, the IgE concentration is regulated by these polymorphisms independently of environmental or lifestyle factors. 

In analogy to the “hygiene hypothesis”, many studies on allergic disease and sensitization observed a protective effect of older siblings (“sibling effect”) [20]. The “hygiene hypothesis” interprets these effects as being the result of a more pronounced exposure to viral and bacterial germs during early childhood and of the correspondingly influenced immune response due to a shift of the immune situation from TH_2_ to TH_1_. The data on the “sibling effect” in eczema are less consistent [20, 21, 22]. In GINIplus and LISAplus, we investigated whether older siblings have a protective effect on eczema development and whether mutations in the filaggrin gene (FLG), which is decisive for an intact skin barrier, modify the “sibling effect”. 

We found that older siblings do not have a protective effect on eczema development. On the contrary, children with the filaggrin mutation had a significantly higher risk of developing eczema if they lived together with older brothers or sisters [23]. 

### Introduction of solid food and development of eczema, asthma, hay fever, and sensitization at the age of 6 years

The WHO recommends exclusively breast-feeding children during their first 6 months of life. It is being discussed whether or not it is reasonable to exclusively breast-feed a child after the 4^th^ month of life. Our studies could not demonstrate that the delayed introduction of solid food beyond the 4^th^ month of life has a protective effect against the development of asthma, hay fever, or sensitization against airborne allergens [24, 25]. For eczema, such a protective effect cannot be excluded. The type and variety of the solid food after the 6^th^ month did not have any effects either [25]. These results have contributed significantly to the simplified new recommendations on solid food in the S3 guideline “Allergy prevention” (AWMF Guideline) [26]. In this context, the most important finding is that the introduction of solid food after the 4^th^ month of life is independent of milk feeding (breast milk or formula); this applies to children with and without allergy risk as long as no signs of an allergic disease are present. 

### Further nutritional factors

We observed that the risk of developing eczema is not influenced by full breast-feeding [27]. Nevertheless, it could be shown that mothers tend to continue full breast-feeding for a longer period of time if their child has skin symptoms. Thus, so-called reverse causation effects cannot be excluded. Breast-feeding mothers did not differ from non-breast feeding mothers or mothers who introduced solid food during the first 4 months with regard to many factors that are relevant for the development of an allergy, such as housing situation, smoking habits, education, household pets, and introduction of solid food. Even in a prospective cohort (as in GINIplus and LISAplus), it is very difficult from a methodological point of view to evaluate the exact role of breast-feeding in atopic diseases. Due to ethical reasons it is not possible to carry out randomized studies on breast-feeding. 

At the age of 2 years, children who mainly eat margarine instead of butter have a higher risk for atopic eczema and allergic sensitization. However, eating margarine seems to be the manifestation of generally different food habits and other lifestyle factors (house pets, smoking, socio-economic status) so that it remains unclear whether margarine and the associated increase in omega-6 fatty acids and linoleic acid is a reason for the development of allergic diseases [28]. 

### Endotoxin exposure and eczema development

Endotoxin is a constituent of the cell membrane of Gram-negative bacteria and can have immunostimulant effects and cause inflammatory reactions. We measured endotoxins in dust samples from the mattresses of 2,000 children and their mothers in Leipzig and Munich when the children were 3 months old. The endotoxin and allergen concentrations in the mother’s mattresses correlated with the cord IgE values. Children in whose mattresses a high content of cat allergen and a moderate content of house dust mite allergen was measured had higher cord IgE values, while higher endotoxin exposures instead led to a reduction of total IgE in the cord blood [7]. Obviously, endotoxins and allergens already influence the child’s immune system and the disposition for allergic reactions *in utero*. Furthermore, a high exposure to endotoxins during the first 6 months of life reduced the risk of developing (unspecific) eczema; a relationship with atopic dermatitis could be demonstrated until the age of 2 years. Also, a longer observation period until the age of 6 years did not show a relationship between the exposure to endotoxins and the development of eczema [29]. 

High endotoxin exposure increases the risk of respiratory symptoms (wheezing) until the age of 2 years. This effect is already detectable at the age of 6 months [30, 31]. 

### Dog and cat as household pets

It is being discussed whether having a dog or cat as a household pet should be recommended as a preventive measure. Data from GINIplus and LISAplus show that children who live together with a dog are less prone to develop an allergy than other children of the same age whose family does not own a dog [32]. A reduced occurrence of allergy was observed for pollen and airborne allergens, while allergy to dog hair occurs neither less nor more frequently. However, the protective effect is limited to children who actually live with a dog; regular contact with dogs alone does not suffice. Why having a dog has a protective effect against allergies has not yet been fully elucidated. Numerous factors could be responsible: a dog-friendly living environment or unknown germs that are brought in by dogs. 

Despite the heterogeneity of the results derived from the different studies analyzed, a systematic review, which also included results from GINIplus and LISAplus, shows that the risk of developing an allergic disease is neither increased nor decreased by keeping a pet [33]. 

### Molds

Various cross-sectional studies have shown that the risk of developing an atopic disease is increased in children who are exposed to molds at home [34]. The chronology of the association between exposure and the occurrence of an allergic disease can only be evaluated by prospective longitudinal studies. A pool of European birth cohort studies aimed at investigating the role of visible mold infestation in households during the first 2 years of life with regard to later occurrence of asthma, hay fever, and eczema. Data from GINIplus and LISAplus were also included. Data derived from a total of 31,742 children from 8 European birth cohorts were evaluated. It was shown that visible mold infestation in a household is associated with a statistically significant increased risk for asthma and allergic rhinitis in school children [35]. 

### Traffic

The analysis of GINIplus and LISAplus data derived from Munich participants showed a relationship between road traffic emissions and asthmatic or allergic diseases as well as between road traffic and allergic sensitization, particularly against outdoor airborne allergens like grass or tree pollen [36]. Asthma and sensitization against pollen increased with elevated exposure to fine dust. 

Increased exposure to nitric oxide was associated with a higher eczema prevalence. The relationship becomes obvious when allergic diseases are regarded in the context of the patient’s living environment; in children who live less than 50 m from a busy street, the risk of developing asthma, hay fever, eczema, or allergic sensitization is increased by up to 50% when compared to their peers who live in streets with less traffic. An analysis of the data derived from the first 2 years of life of children was able to demonstrate a possible relationship between traffic-related air pollution and airway disease [36]. 

LISAplus study results of children from Wesel show that also in smaller towns, like Wesel, traffic-related air pollution can prolong eczematous diseases. In contrast to the Munich results (where a higher level of air pollution is present), no relationship with asthma, hay fever, or allergic sensitization could be established [37]. 

### Indoor exposure

Based on the LISAplus results we could, for the first time, find evidence for the fact that renovation work during pregnancy can influence the child’s immune system. On the basis of immune parameters in the cord blood, we were able to demonstrate functional alterations in the immune system of newborns whose parents carried out renovation work during pregnancy and whose mothers were exposed to increased concentrations of volatile organic compounds (VOC) during pregnancy. The cells that are particularly affected by these alterations are T-lymphocytes. The cord blood of these children contained reduced amounts of TH_1_ cells and increased amounts of TH_2_ cells [38, 39]. As this was associated with an increased risk for the development of atopic eczema during the first 2 years of life 40], it can be assumed that the indoor exposure to chemicals during pregnancy increase the child’s risk of developing an allergy later. 

### Skin symptoms during early childhood and unusual behavior patterns 10 years later

Several cross-sectional studies have demonstrated an association between eczema and mental peculiarities. However, the chronology could not be clarified. The data from the two birth cohort studies GINIplus and LISAplus now make it possible to analyze the association between eczema/skin symptoms during early childhood (first 2 years of life) and the occurrence of unusual behavior patterns almost 10 years later [41, 42]. Both studies showed an approximately 50% increased risk for general behavioral deviations, and particularly for emotional problems, as investigated with a standardized questionnaire (SDQ). The most interesting finding was that this association was also detectable in children in whom the eczema was only present during their first 2 years of life. It has not been clarified whether this association is a causal one because the underlying biologic mechanisms are still unknown. Currently, it can only be speculated if psycho-neuro-endocrine factors could be responsible for both eczema and mental peculiarities. Possibly also sleep disturbances due to pruritus and/or the resulting family interaction play a role in the unusual behavior patterns observed later in life. In this respect, it is also possible that particular behavioral patterns of the parents, which were not investigated in our studies, play a role in the observed association. If the association with the quality of sleep was based on a causal relationship, the successful treatment of atopic eczema during early childhood would have positive long-term effects on the child’s mental health. 

## Prospects 

The 15-year follow-up will not only prolong the observation period but also give insight into the very interesting period of preadolescence and adolescence. During this period, for example, the male dominance in asthma frequency is inverted, and the reason for this is not yet understood. Other possible health-related impairments, like cardiovascular risks, metabolic parameters, and mental health, become more important and will therefore be more intensively studied. 

**Table 1.** Spectrum and instruments of investigation of the birth cohorts GINIplus and LISAplus.

	Age (years)							
	0	1	2	(3)	4	6	10	15
Questionnaires								
Diseases and symptoms (allergies)	X	X	X	X	X	X	X	X
Social factors	X		X			X	X	X
Vaccinations	X	X	X					
Exposure to noxious substances								
Passive smoking	X	X	X	X	X	X	X	X
Indoors	X	X	X	X	X	X	X	X
Outdoors			X			X	X	X
Health-related behavior								
Physical activities Accelerometry						X	X	X X
Nutrition								
Breast-feeding		X	X					
List of items		X	X	X		´X		
FFQ							X	X
Mental deviations							X	X
SDQ							X	X
Depression								X
Kidscreen								X
								
Medical investigation								
Skin			(X)			(X)	(X)	(X)
Lungs						X	(X)	X
FENO							X	X
Anthropometry			X			X	X	X
BIA								X
Blood pressure							X	X
Dental health							(X)	(X)
								
Blood analyses								
Total IgE	(X)	(X)	(X)			X	X	X
Specific IgE	(X)	(X)	(X)			X	X	X
IgA	X		(X)					
Viral AB			(X)					
Vaccination AB			(X)				X	X
T-cell immunity	(X)		(X)		(X)	(X)		
Lipids							X	X
Inflammation markers							X	X
HOMA (fasted blood)							(X)	(X)
Fatty acids	(X)		(X)			(X)	(X)	
								
Genetic analyses (allergies and determinants)						X		
								
Exposure								
Air pollutants (LUR model)	X		X			X	X	X
Noise	X		X			X	X	X
Use of health service / costs							X	X

(X): only in partial samples; FFQ: Food Frequency Questionnaire; SDQ: Strength and Difficulties Questionnaire; FENO: Fractionated Exhaled NO; BIA: bioelectric impedance analysis; AB: antibody; HOMA index: Homeostasis Model Assessment; LUR: Land Use Regression Model.
